# Harnessing Rift Valley fever virus NSs gene for cancer gene therapy

**DOI:** 10.1038/s41417-022-00463-4

**Published:** 2022-04-07

**Authors:** Alicia M. Davis, Tristan A. Scott, Kevin V. Morris

**Affiliations:** 1 Center for Gene Therapy, Beckman Research Institute, City of Hope, Duarte, CA USA; 2 Irell & Manella Graduate School of Biological Sciences, City of Hope, Duarte, CA USA; 3 Menzies Health Institute Queensland, School of Pharmacy and Medical Science Griffith University, Gold Coast Campus, QLD 4222 Brisbane, Australia

**Keywords:** Transfection, Cancer genetics, Cell therapies

## Abstract

One of the greatest challenges in the treatment of cancer is tumor heterogeneity which results in differential responses to chemotherapy and drugs that work through a single pathway. A therapeutic agent that targets cancer cells for death through multiple mechanisms could be advantageous as a broad inhibitor for many types of cancers and the heterogeneous alterations they possess. Several viral proteins have been exploited for antiproliferative and apoptotic effect in cancer cells by disrupting critical survival pathways. Here, we report the use of the non-structural protein on the S segment (NSs) gene from the Rift Valley fever virus (RVFV) to induce cancer cell death. NSs has immune evasion functions in the context of RVFV with many of these functions affecting proliferation pathways and DNA damage signaling, which could be leveraged against cancer cells. We find that expression of NSs in multiple cancer cell lines leads to a rapid decline in cell viability and induction of apoptosis. Interestingly, we observed reduced toxicity in normal cells suggesting cancer cells may be more susceptible to NSs-mediated cell death. To enhance specificity of NSs for use in hepatocellular carcinoma, we incorporated four miR-122 binding sites in the 3’ untranslated region (UTR) of the NSs mRNA to achieve cell type specific expression. Observations presented here collectively suggest that delivery of the NSs gene may provide a unique therapeutic approach in a broad range of cancers.

## Introduction

Cancer remains the leading cause of death worldwide despite over 80 years of dedicated research in cancer drug discovery [1]. There are numerous cancer hallmarks and treatment challenges that have thwarted efforts to eradicate malignancy from patients. One significant challenge in cancer therapeutics is the heterogeneity of genetic alterations that exist in each type of cancer [2]. Tumor heterogeneity fuels resistance to single target drug approaches, which prompts the use of combinatorial therapies [3]. Beyond this, common first-line chemotherapy treatments can result in life-long side effects including neurocognitive defects and cardiotoxicity [4]. For these reasons, physicians and researchers are still exploring novel approaches for the treatment of cancer.

Expression of many plant, fungal, and bacterial toxins have been investigated as anti-cancer therapies based on their ability to slow cancer cell growth or induce apoptosis [5]. There are also numerous viral proteins that have demonstrated anti-cancer activity [6]. The most well known viral protein that has been used for cancer therapy is the Herpes Simplex Virus Thymidine Kinase (HSV-tk) [7]. The HSV-tk protein is used in conjunction with the prodrug ganciclovir and together comprise a suicide gene system that induces cell death through termination of DNA synthesis [8]. Additionally, there are other viral proteins, such as the nonstructural 1 (NS1) protein of parvovirus or the apoptin protein of chicken anemia virus, that disrupt cancer proliferation through multiple essential pathways [6].

Rift Valley fever virus (RVFV) is an arthropod-borne disease causing viral hemorrhagic fever that occurs primarily in livestock but can be transmitted to humans through vectors like mosquitos or contact with blood or organs of infected animals [9]. During RVFV infection, NSs subverts a host immune response by preventing interferon induction [10], suppressing general host RNA pol II transcription [10, 11], degrading dsRNA-dependent protein kinase R (PKR) [11, 12], activating p53 and DNA damage signaling [13, 14], inducing cell cycle arrest [14], and promoting increased reactive oxygen species generation [15]. NSs’ function is restrained during viral replication to ensure cells do not lyse prematurely [16]. However, in the absence of other viral regulators of NSs, its function could be exploited for its anti-proliferative properties for cancer therapy. Based on this rationale, we set out to investigate NSs-mediated cell death as an anti-cancer agent.

In order to introduce exogenous proteins, such as NSs, into mammalian cells they must be delivered by plasmid, mRNA, or as a recombinant protein. Several groups have used nucleic acids to encode toxic proteins and explored their potential as a cancer therapeutic [17, 18]. The expression and activity of suicide genes are generally regulated by delivery vector, promoter, and/or prodrug [19]. Bacterial toxins are often delivered as recombinant proteins tethered to a targeting moiety [17]; however, nucleic acid based expression of toxins is also actively being explored [18, 20]. A plasmid-based approach for delivering diphtheria toxin to cancer cells has been demonstrated in phase 2b clinical trials [21]. However, the use of DNA vectors for gene therapy is not openly accepted due to potential integration into the genome [22, 23]. One alternative to DNA vectors is the use of messenger RNA (mRNA), which displays a short half-life and has no risk of integration. Unlike DNA which must enter the nucleus, mRNA only needs to reach the cytoplasm, which allows for expeditious protein expression and dosing. For these reasons, the use of in vitro transcribed messenger RNA (mRNA) to encode therapeutic proteins is now gaining significant traction [24].

However, the delivery of an mRNA to express a toxic protein in cancer cells should also encode a level of regulation to limit expression in normal cells. One approach to limit mRNA based transgene expression in a certain tissue or cell type is to place microRNA (miRNA) binding sites in the 3’UTR of the mRNA [25]. MiRNAs are small non-coding RNA molecules that post-transcriptionally regulate gene expression through complementary base pairing to the miRNA seed sequence in the target mRNA [26]. MiRNA-based regulation of mRNA has been incorporated for selective self-destruction of cancer cells using endogenous pro-apoptotic proteins such as Puma and Caspase [27]; however, the utility of this regulatory system should be expanded for novel anti-cancer therapies.

In our study, we focused on the development of a novel toxic gene system that is capable of inducing apoptosis in cancer cells in a directed manner and report the development of the NSs gene as a novel anti-cancer nucleic acid therapy. We synthesized an NSs mRNA and delivered it to multiple types of cancer cell lines comprising non-small cell lung cancer, hepatocellular carcinoma, and cervical cancer. We find that NSs leads to a rapid decline in proliferation, viability, and induces apoptosis in all cancer cell lines tested. Additionally, NSs protein expression activated multiple signaling pathways and led to the post-transcriptional degradation of several proteins involved in transcription and response to cellular stress. To mitigate NSs cytotoxic effects in healthy cells, we encoded a miRNA-based regulation cassette in the NSs mRNA to promote cancer specific expression. Together, these data demonstrate a novel approach for inducing apoptosis in cancer cells using the viral NSs protein.

## Results

### NSs reduces viability, proliferation, and induces cytotoxicity in cancer cells

**Fig. 1:**
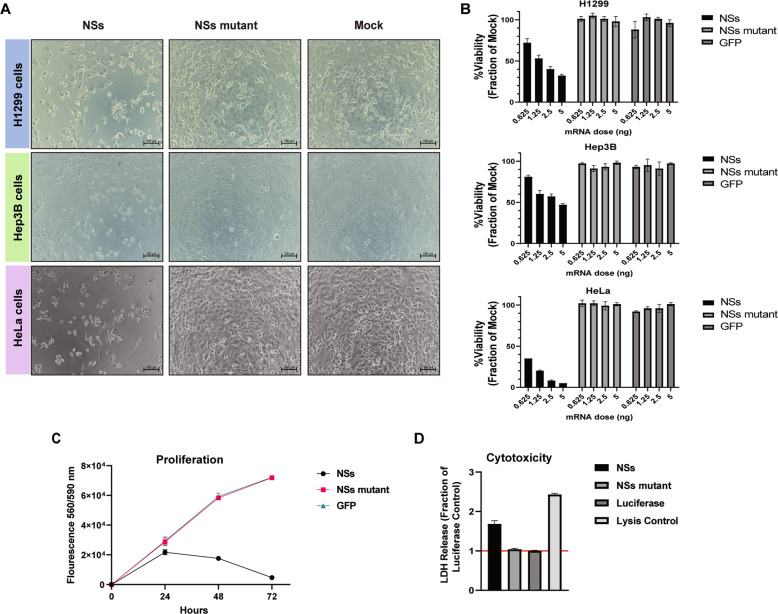
NSs reduces cell viability and induces cytotoxicity in cancer cell lines. **A** Bright field images of cells transfected with 2.5 ng of NSs mRNA, NSs mutant mRNA, GFP mRNA, or mock treatment and captured at 48 h post-transfection. H1299, Hep3B, and HeLa cells were transfected at 2.5 ng mRNA. Scale bar = 100 µm (**B**) AlamarBlue cell viability assays were conducted on H1299 and Hep3B cells transfected with dose titrations ranging from 0.6 to 5 ng of NSs, NSs mutant, or GFP control mRNA at 48 h post-transfection. The treatments were made relative to the mock control set at 100% (**C**) Time course proliferation analysis of H1299 cells transfected with NSs, NSs mutant, or GFP mRNA over 72 h. **D** Cytotox96 assay was performed to measure Lactate Dehydrogenase (LDH) release in H1299 cells transfected with NSs, NSs mutant, or luciferase control mRNA at 48 h post-transfection. A positive control that induced cell lysis was included to demonstrate maximal LDH release. The treatments were made relative to the luciferase mRNA control set at 1. Data presented as the mean ± S.D of triplicate treated cells. These data are one representative experiment that was performed two times.

To investigate NSs-mediated toxicity, NSs mRNA was transfected into cells and cell viability was assessed after 48 h. In the non-small cell lung cancer cell line H1299, NSs expression results in cytotoxicity as visualized by bright field microscopy (Fig. 1A) and reduces cell viability as assessed using the alamarBlue metabolism assay (Fig. 1B). Additionally, we investigated NSs expression in Hep3B (hepatocellular carcinoma) and HeLa (cervical cancer) cell lines, and again observed visual toxicity (Fig. 1A) and a reduction in cell viability (Fig. 1B). We consistently observed both visual cytotoxicity and reduced viability in cancer cell lines transfected with NSs mRNA but not in cells transfected with the GFP control or an NSs mutant (Fig. 1A, B). The NSs mutant contains five amino acid changes (P29A, P32A, P82A, P85A, F261P) within domains critical for NSs function [10, 28]. We next assessed proliferation in H1299 cells over a 72 h time course and found that NSs led to a 3-fold reduction in growth at 48 h and 15-fold reduction at 72 h compared to controls (Fig. 1C). To directly assess NSs-mediated cell toxicity, we measured the amount of Lactate Dehydrogenase (LDH) present in the cell culture media, which is released from cells following plasma membrane damage. Following NSs mRNA transfection, increased LDH release was observed with the NSs mRNA, but not with the NSs mutant or luciferase mRNA controls (Fig. 1D). These data demonstrate that NSs has anti-proliferative and cytotoxic effects on numerous cancer cell lines and may prove useful as a broad anti-cancer gene.

**Fig. 2:**
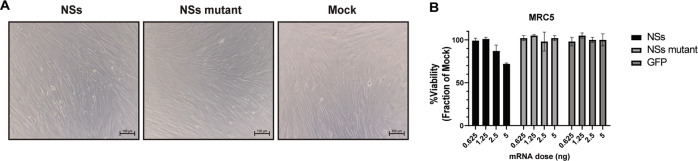
NSs displays reduced toxicity in MRC-5 cells. **A** Bright field images of MRC-5 cells transfected with 2.5 ng of NSs mRNA, NSs mutant mRNA, or mock treatment were captured at 48 h post-transfection. Scale bar = 100 µm. **B** AlamarBlue cell viability assay was conducted on MRC-5 cells transfected with dose titrations ranging from 0.6 to 5 ng of NSs, NSs mutant, or GFP control mRNA at 48 h post-transfection. The treatments were made relative to the mock control set at 100%. Data presented as the mean ± S.D of triplicate treated cells. These data are one representative experiment which was performed two times.

We decided to look beyond cancer cell lines and investigate the effects of NSs in a non-transformed cell. The normal lung fibroblast cell line, MRC-5, has been used for cytotoxicity evaluation of anticancer agents [29, 30]. Interestingly, we did not observe significant visual toxicity during NSs treatment in MRC-5 cells (Fig. 2A). Further, MRC-5 cells appear to maintain higher cell viability when treated with NSs (Fig. 2B). These data suggest that low doses of NSs decrease the viability of cancer cell lines by at least 50% but may have a diminished effect in non-transformed cells.

### NSs induces apoptosis and caspase activation

**Fig. 3:**
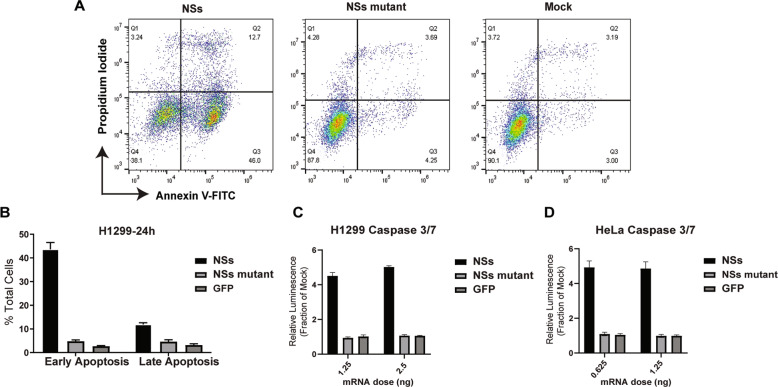
Delivery of NSs mRNA induces apoptosis and caspase cleavage. **A** Representative density plots of propidium Iodide and annexin V-FITC staining of H1299 cells transfected with NSs, NSs mutant, or mock treatment. Evaluation of apoptosis was carried out 24 h post-transfection. **B** Quantification of H1299 cells undergoing early (Annexin V + /PI-) or late (Annexin V + /PI + ) apoptosis. Caspase 3/7 luminescent activity assay was performed in (**C**) H1299 and (**D**) Hela cells at 24 h post-transfection. The treatments were made relative to mock treatment set at 1. Data presented as mean ± S.D of triplicate treated cells. These data are one representative experiment which was performed two times except (**D**), which was performed once.

To determine if NSs causes cells to undergo apoptosis, we used the well-established Annexin-V and propidium iodide flow cytometry analysis [31]. The NSs treated cells rapidly underwent apoptosis after 24 h with 43.6% of cells in early apoptosis (Annexin-V + /PI-) and 11.9% of cells in late apoptosis (Annexin-V + /PI + ) (Fig. 3A, B). To determine if caspase activation is a contributing factor in NSs-mediated apoptosis, a luminescent caspase 3/7 assay was carried out. Indeed, NSs treatment resulted in a 4-fold increase in caspase 3/7 activity compared to the NSs mutant and GFP controls in H1299 (Fig. 3C) and Hela cells (Fig. 3D).

### NSs disrupts transcription and mRNA export

**Fig. 4:**
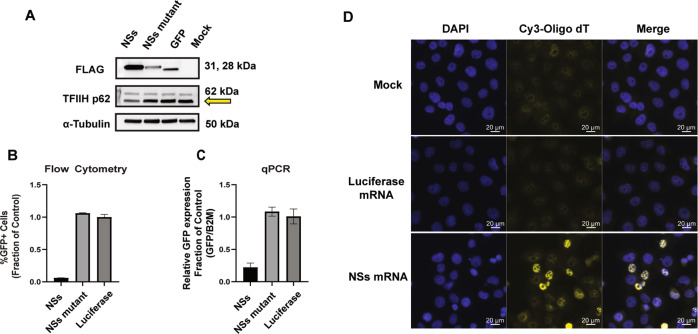
NSs disrupts transcription and mRNA export. **A** Western blot analysis of NSs 3xFLAG tagged protein and TFIIH p62 expression at 24 h post-transfection in H1299 cells. The yellow arrow indicates the TFIIH p62 band. The top band is non-specific antibody binding. **B** Co-transfection of NSs, NSs mutant, or luciferase mRNA with pcDNA-GFP reporter plasmid in H1299 cells. 24 h post-transfection, cells were collected and analyzed for (**B**) GFP expression using flow cytometry and (**C**) RT-qPCR analysis of GFP transcript levels. The treatments were made relative to control mRNA set at 1. Data presented as mean ± S.D of triplicate treated cells. **D** The localization of polyadenylated transcripts in cells was evaluated using an in situ hybridization assay with a Cy3 labeled oligo-dT probe. H1299 cells were transfected with NSs mRNA, luciferase mRNA, or mock treatment and processed for staining post transfection. DAPI (blue) depicts nuclei and Cy3 (yellow) represents polyadenylated transcripts. Scale bar = 20 µm. These data are one representative experiment which was performed two times.

NSs has been linked to various mechanisms in the context of RVFV infection. One mechanism that NSs has been extensively associated with is a shut down of host transcription, which could have anti-proliferative properties. This mechanism is driven in part by the NSs-directed post-translational degradation of the protein p62, which is a critical structural protein in the Transcription factor IIH (TFIIH) complex and RNA pol II transcription [32, 33]. Using H1299 cells, we confirm that NSs protein expression reduced TFIIH p62 protein expression through western blot analysis (Fig. 4A). To investigate the effect of NSs on gene expression, we conducted co-transfection experiments with a pcDNA-GFP expression vector. Through flow cytometry analysis, we observed that NSs suppressed GFP protein expression, with fewer than 6% of total cells expressing GFP (Fig. 4B), and also reduced the level of GFP mRNA expression as evaluated by RT-qPCR (Fig. 4C).

Many bacterial toxins and viral proteins have been linked to inhibition of protein synthesis [18, 34]; therefore, we also investigated the effect of NSs on translation from an mRNA template. As such, we performed co-transfection experiments with a Firefly (Fluc) encoding mRNA to evaluate protein inhibition. However, NSs does not appear to have reduced the level of translation occurring from a reporter mRNA in H1299 (Supplementary Fig. 1A) and HEK293 cells (Supplementary Fig. 1B). Interestingly, NSs has been connected to alterations in mRNA export leading to the accumulation of polyadenylated mRNAs in the nucleus [35]. Indeed, we observed that NSs does contribute to increased accumulation of polyadenylated mRNA in the nucleus as evaluated using fluorescence in situ hybridization with a Cy3 labeled oligo-dT probe (Fig. 4D). These data demonstrate that NSs reduces expression of the TFIIH p62 protein, reduces protein and mRNA expression of a GFP reporter, and leads to the accumulation of polyadenylated mRNAs in the nucleus of cancer cells. These disruptions in critical gene expression pathways may mechanistically contribute to the NSs-mediated toxicity in cancer cells.

### NSs activates p53, DNA damage signaling, and downregulates PKR

**Fig. 5:**
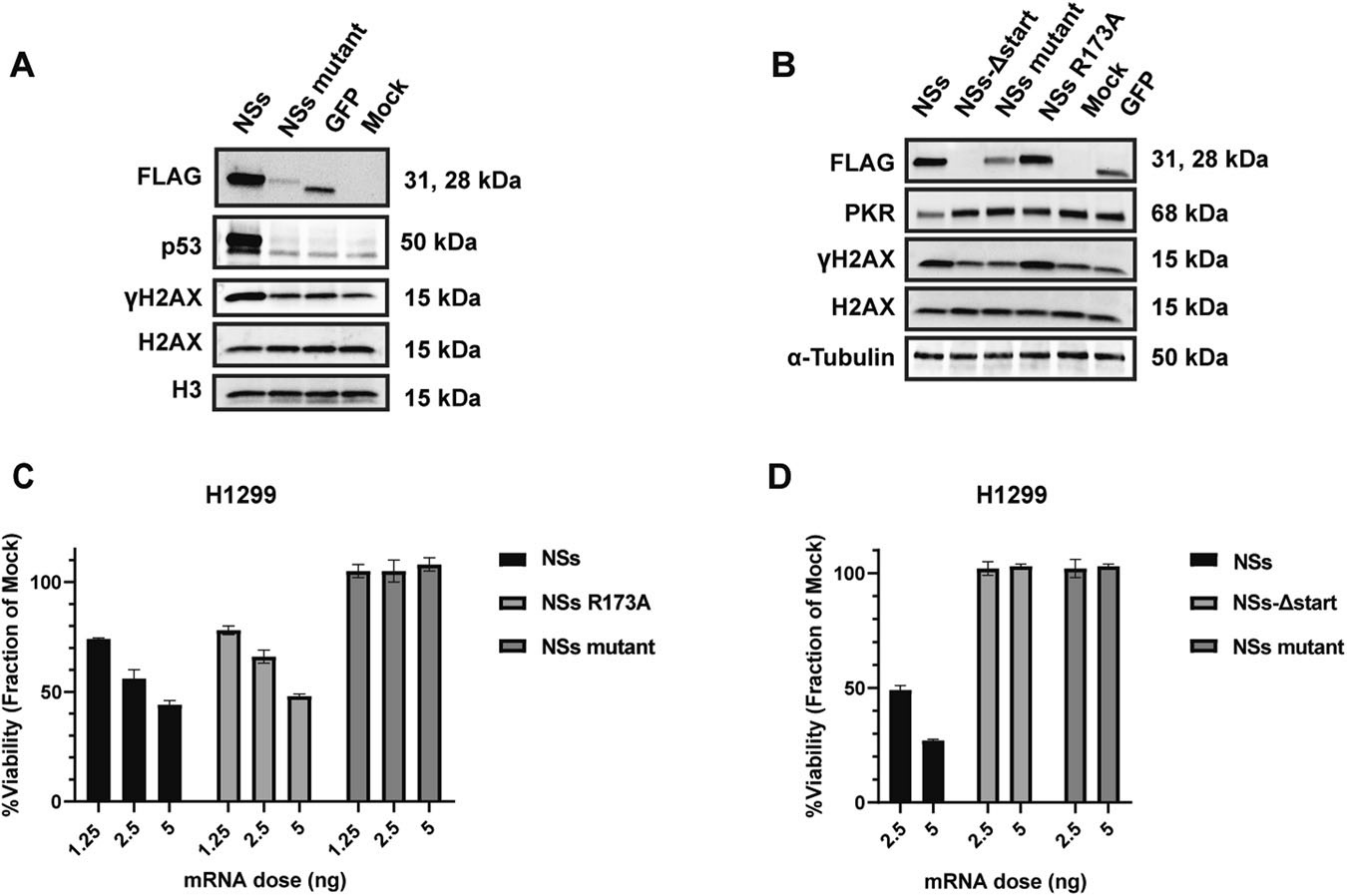
Exploring NSs mechanism in cancer cells using NSs mutants. **A** Western blot analysis to evaluate the induction of p53 and ɣH2AX in A549 cells transfected with NSs, NSs mutant, or GFP mRNA at 24 h post-transfection. H3 was used as a loading control. **B** Western blot analysis of PKR and ɣH2AX in H1299 cells transfected with NSs, NSs-Δstart, NSs mutant, NSs R173A, or GFP mRNA at 24 h post-transfection. **C** Cell viability analysis was assessed at 48 h post-transfection in H1299 cells with NSs, NSs R173A, or NSs mutant. **D** Cell viability analysis was performed at 48 h post-transfection in H1299 cells transfected with either NSs, NSs-Δstart, or NSs mutant. The treatments were made relative to the mock set at 100%. Data presented as mean ± S.D of triplicate treated cells. These data are one representative experiment which was performed two times except (**C**) and (**D**) which were performed once.

Previous work has demonstrated that in the context of RVFV infection, NSs is necessary for the activation of tumor suppressor p53 [13], induction of DNA damage signaling [14], and the post-translational degradation of PKR, which are factors that affect the proliferation and survival of cancer cells [11, 12]. Through western blot analysis, we confirmed that the expression of NSs causes a robust increase in p53 protein expression and this induction is not observed in the NSs mutant or GFP control (Fig. 5A). Furthermore, we observe that NSs expression increases the amount of phosphorylated H2AX protein (ɣH2AX) (Fig. 5A, B), which is the canonical marker of DNA damage signaling [36]. Lastly, we observe a large reduction in PKR protein 24 h after transfection with NSs mRNA (Fig. 5B). To determine if PKR degradation was contributing to the observed NSs toxicity, we cloned an R173A NSs mutant which has been previously shown to lack PKR degradation capacity [37, 38]. We confirm that NSs R173A lacks PKR degradation (Fig. 5B) and observe no substantial difference in toxicity when compared to wild-type NSs (Fig. 5C). Based on these results, we conclude that PKR degradation is likely not a contributing factor to the mechanism of NSs cell death.

### Evaluating the potential role of NSs mRNA sequence in cell toxicity

A lingering question is whether the NSs mRNA itself could be toxic to cells. We used an NSs mutant that contains five amino acid changes (P29A/P32 A/P82A/P85A/F261P) but postulated that altering the sequence at these sites may have changed a non-coding RNA sequence that contributes to toxicity [39]. To test whether the wild type NSs mRNA sequence contributed to toxicity, we mutated the start codon of NSs and synthesized “NSs no start” (NSs-Δstart) mRNA for evaluation. The NSs-Δstart mRNA failed to translate the NSs protein as expected (Fig. 5B) and had no effect on cell viability (Fig. 5D). Together, this demonstrates that the NSs protein is responsible for the observed toxicity and that the RNA sequence does not contribute towards a decline in cell health through a non-coding element.

### Controlling NSs expression using endogenous miRNA binding sites in the 3’UTR

A significant challenge for toxic/suicide gene therapies for cancer is achieving cancer-specific expression. Endogenous miRNA binding sites have been used to prevent transgene expression in unwanted tissues [25], so this approach was explored to control NSs expression in a specific type of cancer [40]. One possible application for NSs is in the treatment of liver cancers such as hepatocellular carcinomas (HCCs), an aggressive group of solid tumors with poor prognosis and treatment options [41]. We explored controlling NSs through the liver specific miR-122 as healthy hepatocytes express high levels of miR-122, but most hepatocellular carcinoma cells lose or have very little miR-122 expressed [42, 43]. In fact, the loss of miR-122 expression in liver cancer has been linked to increased metastatic properties [43]; therefore, targeting these miR-122 negative cells with NSs could eliminate a dangerous population of cancer cells. Based on a previous study [27], we placed four miR-122 miRNA binding sites in the 3’UTR of transgene reporters to test the systems responsiveness to miR-122. Initial testing of the miR-122 system was carried out using an eGFP-ffluc reporter in the presence and absence of miR-122 overexpression from a plasmid (Supplementary Fig. 2A). We observed that with increasing concentrations of miR-122 expression, the luciferase activity of the eGFP-ffluc-4xmiR-122 construct decreased (Supplementary Fig. 2B). We further evaluated the endogenous expression of miR-122 in liver cells using a dual luciferase-based psiCHECK reporter containing miR-122 binding sites. We found that Hep3B cells demonstrated little to no miR-122 activity while Huh7 cells display high miR-122 activity as observed through a reduction in luciferase expression (Supplementary Fig. 2C).

**Fig. 6:**
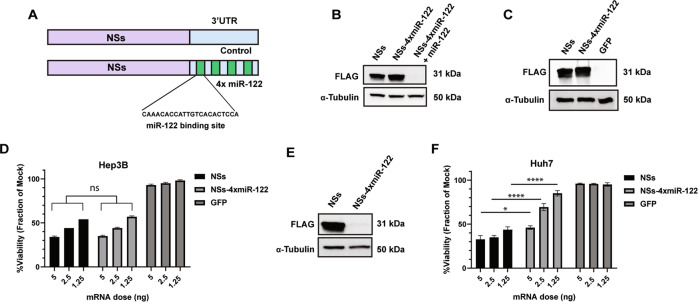
miR-122 regulation of NSs expression in Hepatocellular carcinoma. **A** Design schematic depicting NSs control and NSs-4xmiR-122 mRNA constructs. Four miR-122 target sites were encoded in the 3’UTR of NSs to regulate expression. **B** Western blot expression analysis of NSs, NSs-4xmiR-122, and NSs-4xmiR-122 with miR-122 overexpression evaluated in HEK293 cells at 24 h post-transfection. **C** Western blot analysis of NSs and NSs-4xmiR-122 protein expression in Hep3B cells 24 h post-transfection. **D** Comparing NSs and NSs-4xmiR-122 toxicity in the miR-122 negative cell line Hep3B. Cells were transfected with NSs, NSs-4xmiR-122, or GFP mRNA and cell viability was determined at 48 h post-transfection. Data presented as the mean ± S.D of triplicate treated cells. **E** Western blot analysis of NSs and NSs-4xmiR-122 protein expression in Huh7 cells carried out at 24 h post-transfection. **F** Comparing NSs and NSs-4xmiR-122 toxicity in the miR-122 positive cell line Huh7. Cells were transfected with NSs, NSs-4xmiR-122, or GFP mRNA and cell viability was determined at 48 h post-transfection. The treatments were made relative to the mock set at 100%. Data of three independent experiments presented as mean ± S.E.M.; ns = not significant, *p = 0.01; ****p < 0.0001. A Two-way ANOVA test was performed and corrected using the Tukey method.

Encouraged by these results, the 4xmiR-122 target sites were inserted into the 3’UTR of the NSs coding sequence, termed NSs-4xmiR-122 (Fig. 6A). In the miR-122-negative cell line HEK293, NSs-4xmiR-122 protein expression was unaffected in the absence of miRNA-122 but expression was eliminated in the presence of miR-122 overexpression (Fig. 6B). NSs-4xmiR-122 was tested in the context of hepatocellular carcinoma cells to determine if differential toxicity of NSs could be obtained based on the level of miR-122 expression. We tested the potency of NSs-4xmiR-122 in the miR-122-negative cell line, Hep3B, and found that NSs protein was expressed in this cell line and retained equivalent toxicity compared to WT NSs (Fig. 6C, D). Conversely, in a miR-122-positive cell line, Huh7, the protein expression of NSs-4xmiR-122 was potently reduced at early time points (Supplementary Fig. 2D) and nearly undetectable at 24 h post-transfection (Fig. 6E). In testing NSs-mediated toxicity, NSs-4xmiR-122 showed significantly less toxicity than WT NSs in Huh7 cells (Fig. 6F). Further, NSs-4x-miR-122 displayed very limited effects on cell viability at low concentrations, which shows that incorporating this regulation may be used to modulate NSs-mediated toxicity. Overall, these observations demonstrate that an RNA regulated approach can direct the expression and toxic effects of NSs in a cell type specific manner.

## Discussion

The prospect of developing mRNA therapeutics for numerous diseases is growing rapidly as a result of the massive success of the mRNA based COVID-19 vaccines [44]. Nearly any endogenous or engineered protein can be expressed for therapeutic purposes by delivering a synthetic mRNA. Most mRNA based strategies for cancer focus on a vaccine platform to express cancer antigens [45]; however, there are numerous other research groups intent on providing cancer cells the blueprint for their own destruction in the form of an mRNA encoding a toxic protein [18, 46]. The present study aimed to investigate a novel viral protein, which has been previously shown to act on multiple cellular pathways, as a useful tool for inducing cancer cell death. For this purpose, we used an mRNA to encode the RVFV NSs protein for cytotoxicity testing in cancer cells.

We find that when NSs is introduced into cancer cells as an mRNA, there is a rapid and significant decline in cell health. NSs greatly reduces proliferation and contributes to the release of LDH which is indicative of plasma membrane damage. We confirm that cancer cells are undergoing apoptosis and the induction of robust caspase 3/7 activity at low concentrations of NSs mRNA. Many groups investigating mRNAs encoding a protein capable of inducing apoptosis also include a translation deficient copy of the mRNA to demonstrate that the RNA sequence itself is not contributing to the phenotypes observed [18, 27, 39]. To this end, we tested a translation deficient Δstart NSs mRNA and found that it had no effect on cell viability indicating that the NSs protein and not the RNA is responsible for the cytotoxicity observed. These results demonstrate that the NSs protein is indeed capable of inducing cell death in cancer cells, and we proceeded with studies to understand the cellular alterations that could be driving this enhanced toxicity in cancer cells.

Firstly, we validated that NSs alone leads to the reduction in TFIIH p62 protein expression. Downregulation of p62 has been previously shown to compromise the stability and function of the TFIIH complex; thereby disrupting transcription and also nucleotide excision repair [47, 48]. Based on these previous studies, we hypothesize that NSs-mediated downregulation of TFIIH p62 likely causes the cancer cells to have impaired functionality when challenged with other cellular stresses. Many viral proteins disrupt host transcription or translation in order to favor viral replication [49]; therefore, we explored how NSs alters expression of either DNA or mRNA based reporter systems. We find that NSs alone greatly reduces GFP expression at the protein and transcript level from a DNA template; however, protein expression from a luciferase mRNA is not affected. These results indicate that NSs disrupts gene expression upstream of protein translation. A previous study found that NSs can modulate the export of mRNAs in cells [35], and we also observed accumulation of polyadenylated RNA in the nucleus of cancer cells treated with NSs alone. Beyond NSs, other viral proteins such as HSV-1 virion host shutoff protein and SARS-CoV non-structural protein 1 are also capable of disrupting the export of mRNAs from the nucleus [50]. Importantly, disruption of nuclear export has been highlighted as a broad-spectrum anti-cancer target for drug discovery [51], as cancer cells rely heavily on nuclear export of mRNA to support their proliferation and survival [52]; therefore, NSs likely contributes to a decline in cell health in part through this mechanism.

Many cancer therapies, like chemotherapy, result in the activation of tumor suppressor p53 or the induction of DNA damage to promote apoptosis and elimination of the cell [53, 54]. The activation of these cell signaling pathways also occurs during viral infection. In our study, we observed a strong induction of p53 protein expression and increased DNA damage signaling by the presence of ɣH2AX as a result of NSs expression. Viruses often induce and activate p53 in order to promote their replication and spread [55]. Interestingly we observe NSs cytotoxicity in p53 null (H1299) and wild-type p53 (HeLa) cells indicating that p53 is not solely required for NSs-mediated apoptosis. Furthermore, we observed that NSs increases ɣH2AX expression in H1299 cells. Our results demonstrate that p53 and ɣH2AX induction occur when NSs is expressed alone outside of the context of viral infection. Induction of DNA damage for cancer cell death has been exploited in both chemotherapy and HSV-tk suicide gene systems [8, 54]. Based on our observations, NSs could also serve as a potential DNA damaging agent.

PKR has been identified as a critical kinase in the response to cellular stress [56] and has been highlighted as a potential therapeutic target for cancer due to its role in cancer cell survival and chemosensitivity [57]. We report here that NSs expression alone is sufficient to reduce the amount of PKR expression. However, testing of the R173A NSs mutant deficient in PKR degradation does not alter NSs toxicity, demonstrating PKR reduction is likely not a factor contributing to NSs toxicity in cancer cells. Although, future studies could determine if the NSs-mediated reduction in PKR could sensitize cells to chemotherapy.

Achieving appropriate selectivity towards cancer cell death while limiting normal cell death has proven challenging for nearly all cancer therapies [58]. Importantly, NSs displays high cytotoxicity in cancer cells which likely offers a built in layer of cancer-specific effects. Although we observed less toxicity in a normal non-transformed fibroblast cell line, we encoded an additional layer of protection for normal cells through the miR-122 site and demonstrated selective toxicity in miR-122 expressing cells. However, one challenge with miRNA cassette systems is that a robust toxic protein will likely still have some leaky expression and potentially some toxicity, which was observed in miR-122 positive Huh7 cells at high amounts of NSs-4x-miR122. Notably, Huh7 cells are a cancer cell line and may be more susceptible to any residual expression of NSs than normal liver cells; however, we aim to enhance cancer-selectivity through delivery. Lipid nanoparticles (LNPs) have been successfully used clinically for liver-specific delivery of therapeutic RNAs [59] and alterations to this LNP formulation could be leveraged to increase tumor accumulation and delivery of mRNA [27, 40]. Combining cancer-specific LNP formulations with the engineered miR-122 target site could offer sufficient delivery and expression selectivity for the treatment of liver cancers with NSs mRNA.

In the future, it may be worthwhile investigating an intratumoral administration of NSs to evaluate the anti-tumor efficacy in vivo. If NSs demonstrates good anti-tumor efficacy in the intratumoral model, we propose the incorporation of a tailored lipid nanoparticle formulation to favor distribution to a specific organ where the tumor is established. Lipid nanoparticle formulations containing selective organ targeting (SORT) lipids have unlocked mRNA delivery to the liver, spleen, and lungs in mice [60]. Although these SORT formulations have not been tested in tumor models, achieving organ specific delivery is a great first step that can be combined with miRNA signatures directing the cancer specific expression of mRNA encapsulated in the LNP. Based on this rationale, the liver targeting SORT LNP formulation could be evaluated in a hepatocellular carcinoma model for the delivery of NSs-4x-miR-122 mRNA. Furthermore, antibodies against the epidermal growth factor receptor (EGFR) could be incorporated on the LNP surface to promote receptor mediated endocytosis of LNPs and the release of mRNA cargo in cancer cells [40]. Beyond hepatocellular carcinoma, the lung targeting SORT LNP formulation in combination with an EGFR targeting antibody, and lung cancer specific miRNA signatures could be evaluated for the delivery of NSs. However, due to the high cytotoxic potential of NSs, moving forward with in vivo studies warrants thorough evaluation of off-target toxicity.

Together, the observations presented here show that delivery of NSs mRNA leads to a rapid decline in cancer cell viability and induction of apoptosis through the disruption of multiple pathways which have been highlighted as potential drug targets for cancer. Furthermore, we show that directing NSs expression using miRNA regulatory regions can help reduce potential off-target toxicity. NSs shows great potential as a novel toxic gene and tumor specific expression and delivery strategies may further unlock the utility of NSs for cancer therapy.

## Materials and methods

### Cell culture

The following cell lines were purchased from ATCC and cultured with the indicated medium. NCI-H1299 (ATCC® CRL-5803) (RPMI), HeLa (ATCC® CCL-2) (DMEM), A549 (ATCC® CCL-185)(F12K), MRC-5 (ATCC® CL-171)(EMEM), Hep3B (ATCC® HB-8064) (EMEM), Huh7 (DMEM), 293FT (Invitrogen R70007) (DMEM). RPMI, DMEM, F12K, and EMEM media were all purchased from Gibco (ThermoFisher Scientific, MA, USA) and supplemented with 10% fetal bovine serum (FBS) (GeminiBio CA, USA). Cells were maintained at 37 °C with 5% CO_2_ in a water jacket incubator. All cells were tested for mycoplasma contamination using MycoAlert™ PLUS Mycoplasma Detection Kit (Lonza LT07-710, ME, USA) when thawed from frozen stocks and every 2 months after.

### Plasmids

A complete list of plasmids and the specific cloning strategies used in this study can be found in Supplementary Table 1. All oligonucleotides, ultramers™, and gBlocks® were purchased from Integrated DNA Technologies (IDT; IA, USA). Preparation of DNA inserts was done through either PCR amplification or annealed oligo cloning. PCR amplification of genes was carried out using the indicated forward and reverse primers (Supplementary Table 1) using Q5 HiFi 2X master mix (NEB#M0492S; MA, USA). For site directed mutagenesis, two rounds of PCR were carried out following the procedures outlined in [61]. The resultant amplicons encoded overhangs for use in either restriction enzyme cloning or Gibson assembly as indicated in Supplementary Table 1. Annealed oligo cloning was carried out for generating the miRNA target site constructs. Briefly, ultramer™ oligonucleotides were phosphorylated using T4 PNK (NEB #M0201; MA, USA) according to the manufacturer’s instructions. Complementary ultramer™ strands were annealed at 75 °C for 10 min in a heat block and allowed to slowly cool to room temperature. Preparation of plasmid backbones or PCR products for assembly was carried out using the indicated restriction enzymes (Supplementary Table 1) according to the manufacturer’s instructions (NEB; MA, USA). Assembly of DNA fragments was carried out through either standard T4 ligation or Gibson assembly using T4 DNA ligase (NEB #M0202; MA, USA) or NEBuilder ® HiFi DNA Assembly Master mix (NEB #E2621; MA, USA) respectively, according to the manufacturer’s instructions. The miR-122 expression plasmid was obtained from addgene (Addgene#46673; MA, USA).

### In vitro transcription (IVT) of mRNA

The NSs and GFP DNA templates were prepared for IVT by restriction enzyme digestion with MfeI and XhoI or XbaI (respectively) at cut sites upstream of the T7 transcriptional start site and downstream of the gene of interest stop codon. The digestion products were confirmed on an agarose gel and DNA purified using the Zymo DNA Clean and Concentrator kit (Zymo Research; CA, USA). One microgram of digested template was used in the HiScribe T7 Quick high yield RNA synthesis kit (#E2050; NEB; MA, USA). The transcribed RNA was DNase treated for 15 min at 37 °C and purified using an Invitrogen MegaClear™ kit (Invitrogen CA, USA). The molecular weight of the RNA product was confirmed by gel electrophoresis on a 1.8% agarose/1% bleach TAE gel [62]. The RNA was subsequently capped using the ScriptCap Cap1 Capping System and polyadenylated using the A-Plus Poly(A) Polymerase Tailing Kit according to the manufacturer instructions (CellScript, WI, USA). Successful incorporation of the ~200 nucleotide poly(A) tail was validated by gel electrophoresis. Capped and tailed RNA was treated with Antarctic Phosphatase to remove any remaining 5’ triphosphate groups that could potentially induce immune activation [63]. mRNA was aliquoted and stored at −80 °C until further use.

### Transfection

Plasmid transfection was carried out using Lipofectamine™3000 (Invitrogen 11668019; CA, USA) according to the manufacturer’s guidelines. The mRNA transfection was carried out using Lipofectamine™ MessengerMAX™ (Invitrogen LMRNA001; CA, USA) according to the manufacturer’s guidelines. Cell seeding was performed as follows unless otherwise noted: 96 well:10,000, 24 well: 70,000, 12 well: 120,000, and 6 well: 350,000 cells. The amount of nucleic acid transfected and time points varied based on experiment and are noted in subsequent methods.

### GFP and Luciferase co-transfections

To analyze the effect of NSs on either transcription or translation of a reporter gene co-transfection experiments were carried out. Seventy-thousand cells were seeded in a 24-well plate overnight and transfected with a master mix of lipofectamine™ 3000 and 75 ng of pcDNA3-GFP with approximately 25 ng of NSs, NSs mutant, or luciferase control mRNA. A total of 24 h after transfection cells were collected for flow cytometry and gene expression analysis. Cells collected for flow cytometry were washed twice with PBS and resuspended in 2% FBS-1X PBS. Ten thousand single cell events were collected using a BD C6 Accuri flow cytometer. Data were analyzed using FlowJo Version 10.7.1 (TreeStar; OR, USA). Processing of cells collected for gene expression analyses are described in the “Real-Time qPCR Analysis of Gene Expression” section below.

To analyze the effect of NSs on translation, co-transfection with luciferase mRNA and NSs was performed. Seventy-thousand cells were seeded in a 24-well plate overnight. A transfection master mix of Lipofectamine™ MessengerMAX™ and with 25 ng of luciferase mRNA was prepared to ensure an equivalent amount of luciferase reporter in each condition. This master mix was subsequently incubated with NSs, NSs mutant, or GFP mRNA and added to cells. A 1:1 ratio of reporter: experimental mRNA was used in this experiment because luciferase activity is very sensitive and does not require high input for detection. Media was removed 24 h post-transfection and 100 µl of Glo-Lysis buffer (Promega E2661; WI, USA) was added and placed on a rocker for 5 min. One hundred microliters of lysate was transferred to a white bottom 96-well plate and 100 µl of Bright-Glo reagent (Promega E2610; Madison, WI, USA) was added to each well. Luminescence was measured on a GloMax® Explorer microplate reader (Promega; WI, USA). Relative luminescence was determined by normalizing to the control mRNA sample.

### Dual luciferase assays

To evaluate the endogenous levels of miR-122 expression, 70,000 cells were seeded in a 24-well plate and transfected with 400 ng of psiCHECK2.1 control or psiCHECK2.1 miR-122 using Lipofectamine™ 3000 and a Dual-Luciferase® Reporter assay was carried out according to the manufacturer’s guidelines (Promega E1910; WI, USA). Luminescence was measured on a GloMax Explorer microplate reader (Promega; WI, USA). Data were processed by dividing Rluc/FLuc values and normalized to the psiCHECK control treated cells.

### Viability assays

To assess cell viability, alamarBlue assays were performed. Briefly, 10,000 cells were seeded in a 96-well plate overnight, transfected with an escalating dose of mRNA ranging from 0.625 to 5 ng using Lipofectamine™ MessengerMAX™, and processed at 48 h post-transfection. The alamarBlue assay uses the non-toxic and cell permeable compound resazurin to measure proliferation in cell culture. Resazurin is a colorimetric oxidation-reduction indicator that changes from blue to pink in the presence of metabolically active cells [64]. A 10X Alamar reagent was prepared by mixing 650 µM rezasurin (Sigma Aldrich® 199303, MO, USA), 1 mM potassium ferrocyanide (ACROS Organics™ 196781000, MA USA), 1 mM potassium ferricyanide (ACROS Organics™ 424130050), and 78 µM methylene blue (Sigma Aldrich® M9140) in DPBS (U.S. Pat. No. 5501959). For the assay, the alamar reagent was diluted to 1X in cell culture media and plates were placed on an orbital shaker for 2 min. Plates were subsequently incubated at 37 °C and 5% CO_2_ for 1–2 h. Fluorescence was measured at 520 and 590 nm using the GloMax® Explorer microplate reader (Promega; WI, USA). Blank media values were subtracted from all samples and mock treated cells were used as reference for 100% viability. For multi-day proliferation analysis in Fig. 1C, 5 ng of the indicated mRNAs were transfected into cells seeded in a 96-well plate and alamarBlue readings were done at 24-, 48-, and 72 h post-transfection.

### Bright-field microscopy

In order to capture the visible toxicity, brightfield microscopy images were captured on the ZEISS Axio Vert A1 inverted microscope (ZEISS; Oberkochen, Germany) and processed using ZEN Blue v2.6 software (ZEISS; Oberkochen, Germany). Microscopy images were taken in cells transfected for viability assays described above.

### Cytotoxicity

In order to evaluate cytotoxicity and loss of membrane integrity we performed a CytoTox96 assay (Promega #G1780; WI, USA). Ten-thousand cells were seeded in a 96-well plate overnight, transfected with 10 ng of mRNA using Lipofectamine™ MessengerMAX™, and incubated for 48 h. Prior to beginning the assay, lysis solution (Promega #G1821; WI, USA) at 1X concentration was added to cells for 45 min to serve as a positive control for LDH release. At 48 h post transfection, 50 µl of cell culture media was removed from cells and transferred to a white bottom 96-well plate. Fifty microliters of CytoTox96 reagent was added per well and the plate was rocked gently for 30 min at room temperature. Following incubation, stop solution was added and absorbance was measured at 490 nm using the GloMax Explorer microplate reader (Promega; WI, USA).

### Annexin V/propidium iodide apoptosis analysis

To assess the number of cells undergoing apoptosis or necrosis as a result of NSs, Annexin V and propidium iodide analysis was carried out as described previously [65]. One-hundred thousand cells were seeded in a 12 well plate overnight and transfected with 225 ng of NSs, NSs mutant, or luciferase mRNA using Lipofectamine™MessengerMAX™. And, 24 h post-transfection, cells were gently lifted from the plate using Accutase and were subsequently washed twice with cold 1X PBS. Cells were resuspended in 98 µl of annexin binding buffer, 1 µl of AnnexinV FITC (BD Biosciences Pharmingen; CA, USA), and 1 µl of 1 mg/ml Propidium Iodide (Sigma-Aldrich; MO, USA) and incubated at room temperature for 15 min. An additional 200 µl of annexin binding buffer was added to samples prior to flow cytometry analysis. Ten thousand single cell events were collected using a BD C6 Accuri flow cytometer (BD Biosciences Pharmingen; CA, USA), and the data were analyzed using FlowJo Version 10.7.1 (TreeStar; OR, USA).

### Caspase 3/7 assay

Approximately 10,000 cells were seeded into clear bottom white-walled 96 well plates overnight (Thermo Scientific™ #165306; IL, USA) and were transfected with 0.625, 1.25, or 2.5 ng of NSs, NSs Mutant, or GFP mRNA using Lipofectamine™ MessengerMAX™. At 24 h post-transfection, 100 µl of Caspase 3/7 reagent (Promega G8090; WI, USA) was added to each well and the plate gently rocked for 30 s. Plates were incubated at room temperature for 1 h before detecting luminescence using the GloMax Explorer microplate reader (Promega; WI, USA).

### Western blotting

Approximately 350,000 cells were seeded in a 6-well plate overnight and transfected with approximately 175 ng of mRNA using Lipofectamine™ MessengerMAX™. At 24 h post-transfection, cells were collected and washed once with 1X PBS. Cells were lysed using RIPA buffer (10 mM Tris-HCl pH 8, 1 mM EDTA, 0.5 mM EGTA, 1% Triton X-100, 0.1% sodium deoxycholate, 0.1% SDS, 140 mM NaCl) with 1X Halt protease inhibitor (ThermoFisher Scientific,MA, USA) and sonicated (10 s on 30 s off for 3 rounds) using a Bioruptor 300 (Diagenode; NJ, USA). Soluble protein extract was clarified by high speed centrifugation to pellet debris, and protein concentration was then determined using a BCA Assay kit (PierceTM; ThermoFisher Scientific,MA, USA). Proteins were denatured in 1X Laemmli buffer for 95 °C for 10 min and were electrophoresed on a 4-20% Mini-PROTEAN TGX gel (Bio-Rad; CA, USA) for 25 min at 40 V and then 55 min at 100 V. Thereafter, gels were transferred to nitrocellulose membranes using a semi-dry transfer system (TubroBlot, Bio-Rad; CA, USA), and blocked for 1 h with 3% bovine serum albumin (BSA) dissolved inTBST. Blots were incubated overnight with agitation at 4 °C with a primary antibody solution. The following antibodies were used: M2 FLAG antibody (F3165, Sigma-Aldrich; MO, USA), p53 (sc-126, Santa Cruz Biotechnology; TX, USA), H2AX (2595, Cell Signaling Technologies; MA, USA), ɣH2AX (2577, Cell Signaling Technologies; MA, USA), H3 (4499 S, Cell Signaling Technologies; MA, USA), TFIIH p62 antibody (PA529379, Invitrogen; CA, USA), Alpha Tubulin (ab4074, Abcam; Cambridge, United Kingdom), and GAPDH (sc-47724, Santa Cruz Biotechnology; TX, USA). Thereafter, blots were washed 3X with TBST and incubated for 1 h with a secondary antibody solution. The following secondary antibodies were used: anti-mouse IgG HRP conjugate (1706516, Bio-Rad; CA, USA), anti-rabbit IgG HRP conjugate (1706515, Bio-Rad; CA, USA). Blots were washed as described and were subsequently developed using Pierce ECL2 chemiluminescent reagent (ThermoFisher Scientific; MA, USA) and ChemiDoc Touch imaging system (Bio-Rad; CA, USA). Images were captured based on auto exposure determination within the ChemiDoc Touch system. Images were analyzed and exported to TIFF using Image Lab version 6.1 (Bio-Rad; CA, USA).

### Real-time qPCR analysis of gene expression

To assess changes in gene expression in NSs-treated cells, qPCR analysis was carried out. RNA was isolated using the Maxwell RSC purification kit (Promega; WI, USA), and 10 ng of total RNA was reverse transcribed and amplified using Luna® Universal One-Step RT-qPCR master mix (NEB; MA, USA) in the LightCycler96 real-time PCR system (Roche; Basel, Switzerland). Cycling conditions were as follows: reverse transcription (55 °C for 10 min) and initial denaturation (95 °C for 1 min) followed by 40 cycles of denaturation (95 °C for 10 s) and extension (60 °C for 30 s, with plate read). The fold change in gene expression was determined using the 2-ΔΔCt method. The following qPCR primers were purchased from IDT (Integrated DNA Technology; IA, USA): B2M Fwd: 5’ TAGAGGTGGGGAGCAGAGAA, B2M Rev: 5’ TCCCCCAAATTCTAAGCAGA, GFP Fwd: 5’GACAACCACTACCTGAGCAC, GFP Rev: 5’CAGGACCATGTGATCGCG.

### Microscopy visualization of poly(A) mRNAs

The following methods were adjusted from Copeland et., al 2015 and Kumar and Glausinger 2010 [35, 50]. To visualize the localization of poly(A) mRNAs in NSs treated cells, approximately 27,000 H1299 cells were seeded on poly-L lysine treated cell culture slides (Biologix; MO, USA). After seeding, cells were transfected with 6.75 ng of either NSs or luciferase mRNA using Lipofectamine™ MessengerMAX™. At 24 h post transfection cells were washed once with 1X PBS and then fixed using 4% PFA for 10 min. Cells were permeabilized using ice cold 100% methanol for 5 min and placed in 70% ethanol for 10 min. Cells were washed twice in 1X PBS and incubated in 1 M Tris pH 8 for 5 min. Cells were subsequently incubated for 15 min at 37 °C in prewarmed hybridization solution [10 µg/mL yeast tRNA (Invitrogen; CA, USA), 10% *wt/vol* dextran sulfate (Alfa Aesar;MA, USA), 25% *vol/vol* formamide, 200U of RNAse Out (Invitrogen; CA, USA), and 2X SSC (Sigma-Aldrich; Munich, Germany)]. 5 ng/µL of a Cy3 labeled oligo(dT)_35_ (Integrated DNA Technology; IA, USA) was prepared in the hybridization solution, applied to cells and incubated overnight at 37 °C in the dark. Cells were washed twice with prewarmed 2X SSC, followed by two washes with 0.5x SSC, and incubated in 4% PFA for 10 min. Cells were washed three times with 1X PBS. In order to probe for other intracellular proteins, the slides were blocked using 2% BSA in PBS for 1 h at room temperature. Primary fluorescent antibodies were diluted 1:200 in 0.1% BSA and incubated overnight at 4 °C protected from light. Cells were washed three times with 1X PBS and allowed to air dry. Slides were mounted using Prolong Diamond antifade mountant with DAPI (Molecular Probes; OR, USA) and allowed to set overnight at 4 °C. Slides were imaged using Zeiss Observer Z1 microscope (Zeiss; Oberkochen, Germany). Images were processed using ZEN 2.6 (blue edition) (Zeiss; Oberkochen, Germany).

### Statistical analysis

All data are presented as mean ± S.D. or S.E.M. (unless otherwise noted) and are representative of a minimum of two independent experiments. When appropriate, data were analyzed using two-way ANOVA to compare two or more independent variables. Two-way ANOVA tests were corrected using the Tukey method. *P*-value < 0.05 was considered to be statistically significant. Data were graphed and statistical analyses were performed using Graphpad Prism (version 8.4.3).

## Supplementary information

Supplementary Table 1

Supplementary Figures
