# Supplementary material for: Harnessing Rift Valley fever virus NSs gene for cancer gene therapy
Source: Cancer Gene Ther. 2022 Apr 7;29(10):1477–86. doi: 10.1038/s41417-022-00463-4 (PMC8988100; doi:10.1038/s41417-022-00463-4)
Supplement: Supplementary file 2 — Supplementary Figures [file 41417_2022_463_MOESM2_ESM.pdf]

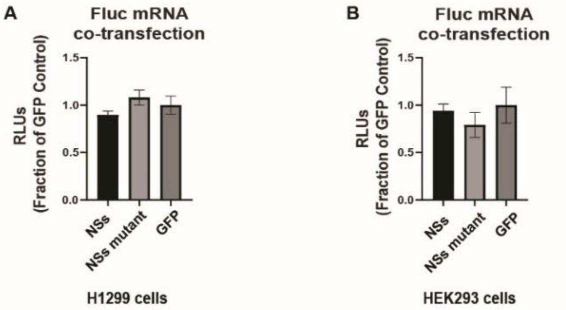

Supplemental Figure 1: NSs does not affect protein translation from an mRNA target. (A) Co-transfection of NSs, NSs mutant, or GFP mRNA with Fluc mRNA reporter in H1299 cells. A Bright-Glo Luminescence assay was performed 24 hours post-transfection. Data presented as mean ± S.D of triplicate treated cells. (B) Co-transfection of NSs, NSs mutant, or GFP mRNA with Fluc mRNA reporter in HEK293 cells. A Bright-Glo Luminescence assay was performed 24 hours post-transfection. The treatments were made relative to GFP control set at 1. Data presented as mean ± S.D of triplicate treated cells. This experiment was conducted once.

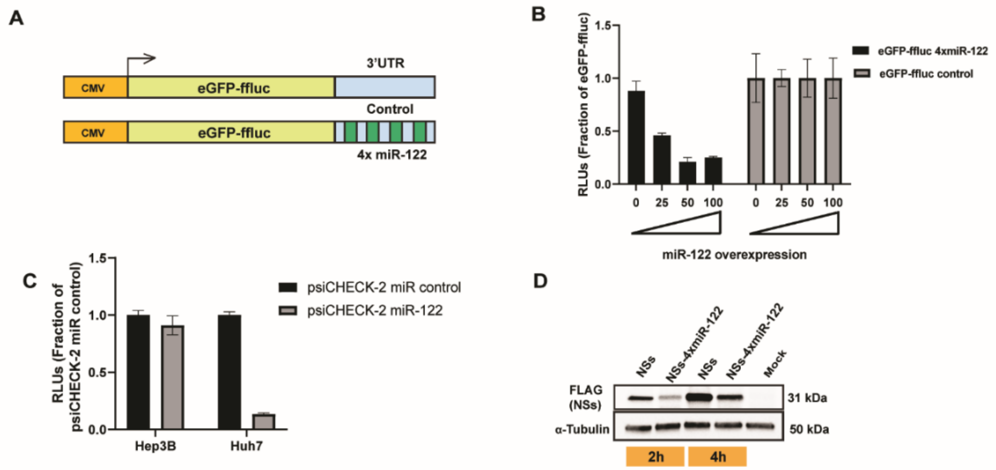

Supplemental Figure 2: Assessing reporter gene activity and protein expression using miR-122 target sites. (A) Design schematic depicting the CMV driven eGFP-ffluc control and eGFP-ffluc4xmiR-122 plasmid reporter containing four copies of the miR-122 target site in the 3'UTR. (B) eGFP-ffluc4xmiR-122 reporter testing was carried out in the presence of increasing concentrations of a miR-122 overexpression plasmid in Hep3B cells at 24 hours post-transfection. (C) The endogenous levels of miR-122 was evaluated using a psiCHECK2.1 luciferase reporter vector containing binding sites for miR-122 downstream of *Renilla* luciferase, or a control vector. The psiCHECK reporters were transfected into either Hep3B or Huh7 cells and luminescence was evaluated using a dual luciferase assay at 24 hours. The treatments were made relative to the indicated control set at 1. Data presented as mean ± S.D of triplicate treated cells. (D) Western blot analysis of NSs and NSs-4xmiR-122 protein expression in Huh7 cells at 2- and 4-hours post-transfection. These control experiments were conducted once.
